# Evolution of the Role of Radiotherapy for Anal Cancer

**DOI:** 10.3390/cancers13061208

**Published:** 2021-03-10

**Authors:** Edward Christopher Dee, James D. Byrne, Jennifer Y. Wo

**Affiliations:** 1 Harvard Medical School, 25 Shattuck St., Boston, MA 02115, USA; Edward_Dee@hms.harvard.edu; 2 Harvard Radiation Oncology Program, Harvard Medical School, Boston, MA 02115, USA; jdbyrne@partners.org; 3 Department of Radiation Oncology, Massachusetts General Hospital, 100 Blossom St., Boston, MA 02114, USA

**Keywords:** chemoradiotherapy, 5-fluorouracil, mitomycin C, intensity-modulated radiation therapy, RTOG 0529, Nigro regimen

## Abstract

**Simple Summary:**

Prior to the 1980s, primary management of localized anal cancer was surgery. Dr. Norman Nigro and colleagues found that neoadjuvant chemoradiotherapy with 5-fluorouracil and mitomycin C afforded complete response, obviating the need for surgery upfront. Advancements in radiotherapy delivery using intensity-modulated radiation therapy (IMRT) and image-guided radiation have resulted in reductions in radiation-associated adverse effects, allowing for the delivery of greater doses of radiation. Ongoing prospective trials are attempting to improve IMRT-based treatment of locally advanced disease with efforts to increase personalized treatment. Trials of newer modalities such as proton therapy are underway. In this review, we present the evolution of radiotherapy for anal cancer and describe recent advances to contextualize ongoing studies and inform future directions in efforts to mitigate treatment toxicities, further personalize treatment, and improve oncologic outcomes.

**Abstract:**

Prior to the 1980s, the primary management of localized anal cancer was surgical resection. Dr. Norman Nigro and colleagues introduced neoadjuvant chemoradiotherapy prior to abdominoperineal resection. Chemoradiotherapy 5-fluorouracil and mitomycin C afforded patients complete pathologic response and obviated the need for upfront surgery. More recent studies have attempted to alter or exclude chemotherapy used in the Nigro regimen to mitigate toxicity, often with worse outcomes. Reductions in acute adverse effects have been associated with marked advancements in radiotherapy delivery using intensity-modulated radiation therapy (IMRT) and image-guidance radiation delivery, resulting in increased tolerance to greater radiation doses. Ongoing trials are attempting to improve IMRT-based treatment of locally advanced disease with efforts to increase personalized treatment. Studies are also examining the role of newer treatment modalities such as proton therapy in treating anal cancer. Here we review the evolution of radiotherapy for anal cancer and describe recent advances. We also elaborate on radiotherapy’s role in locally persistent or recurrent anal cancer.

## 1. Introduction: Surgery Prior to the Introduction of Chemoradiotherapy

An estimated 8590 new cases and 1350 deaths in the United States were attributable to anal cancer in 2020 [1], with increasing incidence in some populations [2]. Human papilloma virus and immunosuppression are two key risk factors for anal cancer [3,4,5,6]. Historically defined prognostic factors for patients with anal cancer include gender, stage, tumor size and nodal status [7]. The presence of human papilloma virus DNA may also be associated with improved outcomes [8]. Recent studies have also suggested low pre-treatment hemoglobin as a poor prognostic factor [9]. Recent studies also suggest immune status–such as the ratio of neutrophils to lymphocytes, the ratio of platelets to lymphocytes, and C-reactive protein–as potential prognostic factors in patients with anal cancer [10].

The primary goal of the treatment of localized anal cancer is locoregional control with the preservation of organ function. Prior to the 1980s, the primary management of localized anal cancer was surgical resection, involving abdominoperineal resection (APR) requiring permanent colostomy [11]. Large surgical series demonstrated 5-year overall survival after APR ranging from 40–70% [12,13,14,15,16]. Abdominoperineal resection also yielded significant morbidity due to lack of sphincter preservation and high rates of sexual and urinary dysfunction [14,17,18].

In an effort to improve operability, Norman Nigro and colleagues at Wayne State University attempted neoadjuvant chemoradiotherapy; their early experience demonstrated a complete response in the first three patients treated with chemoradiotherapy, published in 1974 [19]. Subsequent studies supported local excision instead of APR, and eventually, nonsurgical primary management [19,20,21].

The Nigro regimen used 30 Gray (Gy) of external beam radiation therapy with mitomycin C (MMC; 10–15 mg/m^2^ on day 1) and 2 cycles of 5-fluorouracil (5FU; 1000 mg/m^2^ on days 1–4 and days 29–32), achieving a complete response in the first three treated patients [19]. In a follow-up study with 45 patients, 38 (84%) patients were rendered free of disease with nonsurgical management; Leichman et al. wrote: “It appears that the adjuvant chemotherapy does not merely ‘sensitize’ the local tumor for the radiation therapy but, in combination with radiation, ‘kills’ local tumor” [22]. The study also demonstrated a 5-year overall survival of 67% and colostomy-free survival of 59% [22].

Seminal work from Jean Papillon published in the 1980s also supported the establishment of nonsurgical treatment of anal cancer [23,24,25]. A series of 121 patients with anal cancer treated with radiation, with or without subsequent surgery, the rate of cancer-specific mortality was 18% [24]. Of patients for whom treatment was curative, three-quarters maintained normal anal function [24]. Another series of 221 patients treated with external beam radiation to 30 Gy and iridium implant boost to 10–20 Gy resulted in a 5-year overall survival rate of 65%, 90% of whom maintained normal anal function [23]. In a subsequent series, 222 patients were treated with external beam irradiation followed by iridium implant, 80% of patients experienced tumor control and 90% retained normal anal function [25]. The authors also suggested “chemotherapy during the first days of irradiation…to reinforce the efficacy of treatment and increase the chance of anal preservation” [25].

Although APR remained an option for patients with persistent or recurrent disease, with a 5-year survival rate up to 92% [20,22,26,27,28,29,30,31,32,33,34,35,36,37,38,39], subsequent studies assessing primary treatment options for localized anal cancer sought to optimize upfront nonsurgical management.

## 2. Chemoradiotherapy versus Radiotherapy Alone: ACT I and EORTC

In light of adverse effects associated with concurrent 5FU and MMC with radiation therapy, other studies in the 1980s provided evidence that radiotherapy alone produced promising outcomes [40,41]. A key retrospective series demonstrated 3-year overall survival of 75% for patients receiving radiotherapy alone [40,41]. Another series demonstrated 5-year overall survival rates upwards of 60–70% with radiation alone, with maintenance of sphincter function in the vast majority of patients [23]. The success of regimens consisting of radiotherapy alone called into question the role of chemoradiotherapy in the 1980s [42]. However, non-retrospective evidence comparing chemoradiotherapy vs. radiation alone suggested improved local control rates for chemoradiotherapy [43]. Therefore, two randomized controlled trials were proposed to compare head-to-head chemoradiotherapy vs. radiotherapy alone.

In the 1996 United Kingdom Coordinating Committee on Cancer Research (UKCCCR) Anal Cancer Trial (ACT I) trial, 585 patients were randomized to radiotherapy alone (45 Gy with external beam radiotherapy (EBRT) and either 15 Gy EBRT boost or 25 Gy brachytherapy boost) or chemoradiotherapy (similarly 45 Gy with EBRT and either 15 Gy EBRT boost or 25 Gy brachytherapy boost, with concurrent MMC at 12 mg/m [2]. on day 1 and 5FU at 1000 mg/m^2^ for 4 days or 750 mg/m^2^ for 5 days during the first and last week of radiation) [44]. The study found no significant difference in 3-year overall survival between chemoradiotherapy and radiotherapy (65 vs. 58%, *p* = 0.25); however, 5-year local control for chemoradiotherapy was significantly greater than for radiotherapy alone (68 vs. 43%, *p* < 0.001) [44,45].

The 1997 European Organization for Research and Treatment of Cancer (EORTC) trial randomized 110 patients to radiation therapy (45 Gy EBRT with 15 Gy or 30 Gy EBRT boost) or chemoradiotherapy (45 Gy EBRT with 15 Gy or 30 Gy EBRT boost with concurrent 5FU at 750 mg/m^2^ continuous infusion on days 1–5 and 29–33 and MMC at 15 mg/m^2^ one day 1) [46]. The study found no significant difference in 3-year overall survival between groups (65 vs. 72%, *p* = 0.17); however, the colostomy-free survival was 72% for patients who received chemoradiotherapy vs. 40% for patients treated with radiotherapy alone [46]. Notably, chemoradiotherapy was associated with improved 5-year local control compared with radiotherapy alone (68 vs. 51%, *p* = 0.02) [46].

The UKCCCR/ACT1 and EORTC trials were similar in their use of 45 Gy radiotherapy and comparable doses of concurrent 5FU and MMC, their 6-week post-treatment break and radiotherapy boost to patients with partial and complete response, and definitive surgery after the 6-week break for nonresponsive disease [44,46]. Both trials were also similar in their demonstration of improved local control and colostomy-free survival in the chemoradiotherapy arm [44,46]. Long-term 13-year follow-up of the UKCCCR/ACT1 trial also demonstrated significantly lower risk of cancer-specific mortality in the group that received combined therapy [45]. However, for both trials acute toxicities were greater in the chemoradiotherapy arm, late toxicities were similar between treatment groups [44,45,46,47].

Taken together, the UKCCCR/ACT1 and EORTC trials in the late 1990s support the role of chemoradiotherapy compared to radiotherapy alone in providing superior control of local disease, colostomy-free survival, and disease-specific survival [44,46]. The lack of difference in overall survival has been attributed to the role of salvage APR–despite surgical morbidity–in controlling and eliminating persistent or recurrent disease. These studies established chemoradiotherapy as standard-of-care for anal cancer. Key completed trials are summarized in Table 1.

## 3. Omission of Mitomycin C: RTOG 87-04/ECOG 1289

The Radiation Therapy Oncology Group (RTOG) and Eastern Cooperative Oncology Group (ECOG) RTOG 87-04/ECOG 1289 trial evaluated the omission of mitomycin C (MMC) in concurrent chemoradiotherapy for patients with anal cancer. The inclusion of MMC in the Nigro protocol likely contributes to the majority of the adverse effects experienced by patients on the Nigro protocol and is associated with leukopenia, thrombocytopenia, nephrotoxicity, and pulmonary toxicity [47]. RTOG 87-04/ECOG 1289 randomized 310 patients to radiation (45–50.5 Gy) with 5FU alone (1000 mg/m^2^ on days 1–4 and 29–32) vs. 5FU with MMC (10 mg/m^2^ for two doses) [48]. The RTOG 87-04/ECOG 1289 trial demonstrated an association between the addition of MMC and higher colostomy-free survival (71% for 5FU + MMC vs. 59% for 5FU alone; *p* = 0.014); the trial also demonstrated an association between MMC and improved disease-free survival (73% for 5FU and MMC vs. 51% for 5FU alone; *p* = 0.0003). Although the addition of MMC did not improve overall survival and anal cancer-specific survival and was associated with greater toxicity (23 vs. 7% experiencing grade 4 or 5 toxicity, *p* < 0.001) [48], the results of RTOG 87-04/ECOG 1289 support continued inclusion of MMC in the treatment of anal cancer.

## 4. Cisplatin as a Possible Alternative to Mitomycin C: RTOG 98-11 and ACT II

Due to cisplatin’s efficacy in certain squamous cell cancers [56] and early studies demonstrating promising results in anal cancer [39,57,58], RTOG 98-11 prospectively assessed cisplatin as a substitution for MMC. RTOG 98-11 randomizes 682 patients to either radiation with 5FU (1000 mg/m^2^ on days 1–4 and 29–32) and MMC (10 mg/m^2^ on days 1 and 29), or induction chemotherapy with 5FU (1000 mg/m^2^ on days 1–4 and 29–32) and cisplatin (75 mg/m^2^ on days 1 and 29) followed by chemoradiotherapy with 5FU and cisplatin (starting day 57). The primary tumor and perirectal nodes were treated to a minimum of 45 Gy in 25 fractions once a day, with advanced disease receiving an additional boost to 10–14 Gy. The initial publication found lower rates of colostomy in patients receiving MMC vs. cisplatin (10 vs. 19%, *p* = 0.02), although MMC was associated with greater toxicity; there was no difference in disease-free survival and overall survival at 5 years [49]. A subsequent publication of RTOG 98-11 demonstrated improved 5-year disease-free survival (67.8 vs. 57.8%, *p* = 0.006) and 5-year overall survival (78.3 vs. 70.7%; *p* = 0.026) in the MMC arm [7,59] The MMC arm also demonstrated nominally lower rates of local recurrence (20 vs. 26.4%, *p* = 0.087) and colostomy (11.9 vs. 17.3%, *p* = 0.074) [7,59] Of note, pooled analysis of RTOG 87-04/ECOG 1289 and RTOG 98-11 demonstrated an association between longer treatment time and increased colostomy failure, greater local failure rates, and worse disease-free survival [60], possibly introducing bias due to longer treatment time in the RTOG 98-11 cisplatin arm.

The ACT II trial assessed whether substituting cisplatin for MMC and whether the inclusion of maintenance chemotherapy improved outcomes in anal cancer [50]. Patients were randomized in a 2 × 2 fashion to F5U with MMC vs. 5FU with cisplatin, and observation vs. maintenance chemotherapy with 2 cycles of 5FU/cisplatin. At 6 months, rates of clinical complete response and grade 3–4 toxicity were similar between the MMC and cisplatin arms. Three-year rates of progression-free survival were similar in the maintenance vs. observation arms (74 vs. 73%, *p* = 0.70) [50].

ACT II demonstrated that cisplatin and maintenance chemotherapy were not superior to standard-of-care. Furthermore, cisplatin administration requires intravenous hydration and greater anti-emetic therapy. Therefore, 5FU with MMC remain standard of care.

Of note, although trials in Europe have tended to use one cycle of MMC and trials in the US have tended to use two cycles, retrospective data comparing 5FU with one or two cycles of MMC found no difference in progression-free survival, cancers-specific survival, overall survival, and colostomy-free survival between both arms, with lower toxicity in patients receiving one cycle of MMC [61].

## 5. Capecitabine as an Alternative to 5FU

Capecitabine has been investigated as an alternative to 5FU for anal cancer [62]. Capecitabine’s relative ease of administration and potential radiosensitizer properties prompted further study of its role in anal cancer [62]. A phase II trial evaluated capecitabine 825 mg/m^2^ twice daily and MMC 15 mg/m^2^ on day 1 during radiation therapy and demonstrate a complete response rate at 6 months of 86%; 7% of patients experienced partial response, and 7% experienced disease progression [63]. In another phase II trial of capecitabine and MMC with radiotherapy, 77% of patients experienced complete response, 16% experienced partial response, and 7% experienced disease progression; 14% experienced locoregional relapse [64].

The role of capecitabine as an alternative to 5FU has also been supported by the experience in rectal cancer from the National Surgical Adjuvant Breast and Bowel Project R-04 (NSABP R-04) [65]. NSABP R-04 was a 2 × 2 trial that randomized 1608 patients with stage II or III rectal cancer to radiotherapy with continuous venous infusion 5FU or oral capecitabine, with or without oxaliplatin [65]. Patients treated with capecitabine compared to those treated with 5FU did not experience a significant difference in surgical downstaging, sphincter-sparing surgery, and pathologic complete response, lending support to the role of capecitabine in anal cancer [66].

A retrospective study of UK patients comparing toxicity experienced by patients treated with capecitabine/MMC vs. 5FU/MMC found similar rates of grade ≥3 toxicity between groups (capecitabine/MMC 45 vs. 5FU/MMC 55%, *p* = 0.35) [67]. Furthermore, the rate of grade 3 hematologic toxicity was lower for patients who received capecitabine/MMC vs. 5FU/MMC (4 vs. 27%, *p* = 0.001) [67]. However, the lack of randomized phase III data specific to anal cancer has limited the universal adoption of capecitabine. Also, concerns remain regarding treatment adherence as treatment typically involves multiple tablets taken twice daily.

## 6. Radiation Dose De-Escalation

Given the risk of acute and late adverse effects from large radiation doses, de-escalation is also under investigation. The series presented in 1985 by Leichman et al. showed that for patients with T1-T2 tumors with close or involved margins treated with chemoradiotherapy, doses as low as 30 Gy could achieve local control rates of 90% [22]. However, more recent retrospective studies suggest that doses <50 Gy are associated with greater rates of local failure compared to doses ≥54 Gy, although variation in tumor and patient characteristics as well as different types of chemotherapy and radiation field employed complicates the interpretation of these studies [57,68,69,70,71]. Given the variation in local control rates by tumor size and potential decrease in adverse effects with de-escalation [72,73,74], the ongoing ECOG-DECREASE study aims to assess the role of dose de-escalated chemoradiation for early stage anal cancer [75]. Patients will be randomized to standard-dose chemoradiation (28 fractions with MMC and either capecitabine or 5FU) or de-intensified chemoradiation (20–23 fractions with MMC and either capecitabine or 5FU). Primary endpoints include disease control (maintenance of 2-year disease control of 85% or higher in the dose de-escalated cohort) and health-related quality of life. [75]. The ongoing ACT III and ACT IV trials, discussed further below, will assess dose de-escalation in patients with T1-T2 disease [76].

## 7. Radiation Dose Escalation

The role of dose escalation has also been investigated. In the earlier RTOG 92-08 trial, a total dose of 59.4 Gy was delivered in 1.8 Gy fractions with a 2-week treatment break [36,48,51]. Patients treated with this higher dose had greater colostomy rates of 30%, compared to 9% in RTOG 87-04, where patients received 45 Gy without a treatment break and similar chemotherapy as in RTOG 92-08 [36,48,51]. Late effects of dose escalation were not reported in the initial publication and subsequent updates [36,48,51].

However, data supporting variations in outcome by tumor size galvanized further investigation of dose escalation [72,73]. An analysis of RTOG 98-11 demonstrated that tumors with diameter >5 cm were associated with lower 5-year disease-free survival and overall survival [72]. Another re-analysis of RTOG 98-11 found that 3-year colostomy failure rates differed by tumor size, with failure rates of 28% for patients with T4N1-3 disease vs. 4% for patients with T2N1-3 disease [73]. Therefore, investigators explored the use of adapted radiation dose escalation.

The ACCORD-03 trial was designed to assess high-dose RT boost based on initial treatment response [52]. In ACCORD-03, patients who responded to initial chemoradiotherapy received standard 15 Gy boost or dose-escalated 20–25 Gy boost (patients with complete response or >80% tumor reduction received 20 Gy; the rest received 25 Gy). The dose-escalated boost arm was treated with EBRT or LDR brachytherapy [52]. The trial found no difference in the primary endpoint of colostomy-free survival (78 vs. 74%, *p* = 0.067). However, ACCORD-03 demonstrated improved 5-year local control for the group treated with dose-escalated boost (83.1%) vs. standard 15 Gy boost (78.2%), although this difference was not significant [52]. Of note, since patients had a 3-week break prior to the boost in addition to induction chemotherapy, the possible association between increased overall treatment time and accelerated tumor repopulation [68] may have contributed to the study’s findings.

A concern for dose escalation includes an increase in the risk of fecal incontinence [74]. A European study found that in a cohort treated with 56 Gy, one-third of patients experienced fecal incontinence [74,77,78]. Very high doses delivered to the lamina propria and anal sphincter may also result in stricture and stenosis. Therefore, efforts are needed to balance these risks with potential improvements in local control.

In the era of IMRT, dose-escalation continues to be under investigation for patients with locally advanced disease [79]. A 2014 review of IMRT developed a linear quadratic dose-response model, suggesting that for IMRT-treated patients with anal cancer, a >5 Gy increase in dose may improve local control rates by >10% [80]. Despite potential gains in treatment efficacy associated with dose escalation, increased doses must still be weighed against late toxicities of higher doses [74]. The ongoing ACT V trial, discussed later in this review, will assess dose escalation for patients with T3-T4 disease [76].

## 8. Intensity-Modulated Radiation Therapy: RTOG 05-29

**Figure 1:**
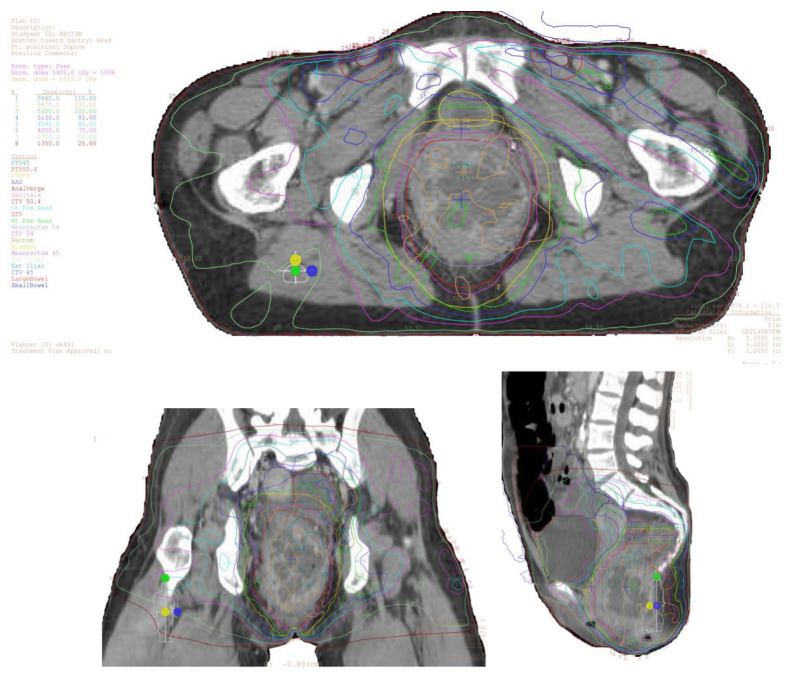
Example of 3DCRT photon plan for a patient with localized anal cancer. The primary tumor involved lymph nodes, and elective nodes were treated to 54 Gy, 50.4 Gy, and 45 Gy in 30 fractions, respectively.

Earlier randomized trials for anal cancer (ACTI to ACT II) relied on older radiation techniques using 2–4 fields that provide substantial radiation doses to organs at risk nearby the target volume [47]. IMRT, which modulates radiation intensity in a given field often with nine or more fields, allows delivery of more conformal dose, facilitating coverage of the clinical target with reduction of dose to surrounding normal organs at risk, possibly resulting in fewer treatment breaks and decreased overall treatment time [53,68]. Various retrospective and small prospective series demonstrated reductions in gastrointestinal and skin toxicity with the use of IMRT relative to the MMC arm of RTOG 98-11, where patients were treated with 2-dimensional or 3-dimensional conformal radiotherapy (3DCRT, Figure 1) [79,81,82,83,84,85,86,87,88,89,90,91,92]. Several institutional series compared patients treated with 3DCRT and IMRT and also demonstrated improved dermatologic and gastrointestinal toxicities, with comparable disease-specific outcomes compared to the MMC arm of RTOG 98-11 [85,93,94,95,96,97,98].

Therefore, concurrent dose-painted IMRT and 5FU/MMC with chemotherapy was prospectively evaluated with RTOG 05-29 [53]. The study’s primary endpoint was a reduction of 15% in gastrointestinal and genitourinary toxicities compared to the treatment strategy employed in RTOG 98-11 [7,53,59]. Patients in RTOG 05-29 with T2N0 disease received 50.4 Gy tumor PTVs and 45 Gy elective nodal irradiation; patients with T3-4N0-3 disease received 54 Gy to the tumor PTV, 50.4 Gy for <3 cm metastatic nodes or 54 Gy for >3 cm metastatic nodes, and 45 Gy to electively treated nodes [53].

Dose-painted IMRT was associated with reduced 3+ gastrointestinal and genitourinary toxicities (22 vs. 36%, *p* = 0.014) and reduced grade 3+ dermatologic toxicity (20 vs. 47%, *p* < 0.001) compared to the MMC arm in RTOG 98-11. Patients in RTOG 05-29 also had shorter median treatment duration (43 days vs. 49 days, *p* < 0.001) and shorter median duration of toxicity-related treatment breaks (0 vs. 3 days, *p* < 0.001) compared to historical controls.

As RTOG 05-29 assessed treatment toxicity as a primary endpoint, two recent series from the US and Italy report oncologic outcomes experienced by patients treated per RTOG 05-29 [99,100]. A US series of 99 patients with dose-painted IMRT according to RTOG 0529 dosing guidelines and found that at a median follow-up of 49 months, 92% of the cohort experienced a clinical complete response [99]. Of these 99 patients, 15 underwent colostomy, 11 underwent APRs, and 13 eventually developed local recurrence; overall survival at 4 years was 85.8%, and event-free survival at 4 years was 75.5% [99]. Overall, 20% of patients experienced grade 3 acute and 15% experienced grade 2+ late toxicities [99].

Similarly, a study in Italy reported outcomes of 87 patients treated according to the RTOG 05-29 protocol [100]. At 3 years, rates of local control, disease-free survival, and overall survival were 69%, 71%, and 79%, respectively; 15.1% underwent colostomy at 24 months and 16.4% experienced cancer-specific mortality at 36 months [100]. Another European multicenter series retrospectively assessed 190 patients with anal cancer who received concurrent chemoradiation with either simultaneous integrated boost IMRT or 3D conformal RT and sequential boost, which involved progressive boost to selected target regions with macroscopic disease [101]. There were no significant differences in cancer-free survival and overall survival between groups [101]. Along with RTOG 05-29, these findings established IMRT in the treatment of anal cancer.

## 9. Improvements in Radiotherapy Simulation

Radiation treatment simulation should involve attention to a patient’s prior rectal, gynecologic, and inguinal node exams, as well as careful consideration of a patient’s imaging and patient factors such as comfort to treatment position [68]. Although the prone position on a belly board is preferred due to bowel sparing, it comes at the cost of inter-treatment variation and may be affected by the patient’s anatomy and degree of bladder filling [102]. Distention of the bladder may be employed with the belly board to facilitate bowel sparing [103]. Immobilization devices such as Vac-Lok cradles, vaginal dilators, and anal markers or wires may also improve simulation and reproducibility [104]. Furthermore, the use of bolus for patients with peri-anal skin involvement, as was done in RTOG 05-29 [53], may be obviated if techniques such as volumetric arc therapy (VMAT) are used [105]. Intravenous and oral contrast may also aid the delineation of organs at risk [106].

## 10. Treatment Planning and Delivery: Institutional Practice and Recent Advances

The use of PET-CT and MRI-CT fusion-based planning have allowed improvements in target delineation and avoidance of organs at risk [107]. RTOG 05-29, which established the feasibility of IMRT, was designed with generous clinical and planning target expansion to ensure adequate tumor coverage [53]. At our institution, image-guided radiation therapy often allows the use of tighter margins. We employ CTV of the primary tumor and anal canal with 1.0–1.5 cm margins radially and 2.5 cm margins superiorly. We also use a 0.7 cm PTV expansion.

Per RTOG guidelines, radiation doses are based on patients’ staging: patients with T2N0 disease received 42 Gy elective nodal and 50.4 Gy anal tumor PTVs in 28 fractions, and patients with T3-4N0-3 disease received 45 Gy elective nodal and 50.4 Gy <3 cm or 54 Gy >3 cm metastatic nodal and 54 Gy anal tumor PTVs in 30 fractions. Although data from other institutions make use of sequential boost strategies with IMRT doses up to 59.4 as in RTOG 98-11 [49], at our institution, patients are treated with dose-painted IMRT in accordance with RTOG 05-29.

At our institution, inguinal lymph node biopsies are not routinely used to pathologically assess nodal involvement. If nodes demonstrate tumor involvement on PET/CT or other imaging, clinically involved nodes are treated to 50.4 Gy for nodes <3 cm or to 54 Gy for nodes >3 cm. Although some studies, including RTOG 05-29 recommend radiating involved nodal regions to 50.4 Gy and 54 Gy, our institutional practice treats uninvolved nodes electively to 45 Gy.

The development of various contouring atlases has also aided the standardization of contouring techniques [108,109,110,111]. Amongst normal structures, we recommend contouring large bowel, small bowel, femoral heads, iliac bones, bladder, perianal skin, and genitalia. We recommend maximizing PTV with >90% of the primary tumor and involved nodes receiving prescription dose coverage, and with >85% of elective nodal PTVs receiving the prescription dose. We recommend correction for tissue heterogeneity. Treatment is delivered once daily with five fractions per week, with daily image guidance for prone treatment delivery.

Although our institution uses RTOG 05-29 dose constraints, adequate target coverage is often challenging without exceeding OAR dose-volume constraints. Doing so is particularly difficult for patients with large tumors or patients with large proportions of small bowel in the treatment field.

**Figure 2:**
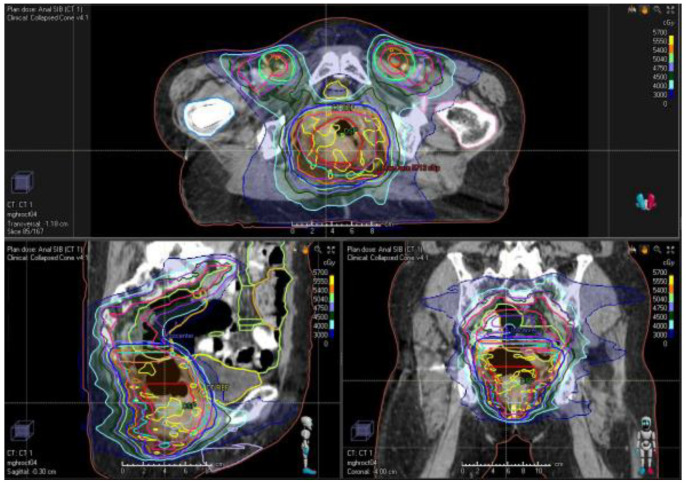
Example of VMAT photon plan for a patient with clinically staged T2N1 anal cancer. The primary tumor and mesorectum, involved lymph node, and elective nodes were treated to 54 Gy, 50.4 Gy, and 45 Gy in 30 fractions, respectively.

Improvements in treatment may also be afforded by VMAT, image-guided, MRI-based, and adaptive planning. Rotational techniques such as VMAT reduce treatment time compared to static IMRT (Figure 2) [112,113,114,115,116]. IMRT and image-guided radiotherapy with daily imaging and adaptive planning may also decrease the dose delivered to major OARs [68,112]. Advances in adaptive planning, involving adjustments of radiation plan based on patient-specific changes during the course of treatment, make use of cone-beam CT adjusted as the tumor shrinks. PET- and MRI-based planning may improve target delineation, although in our institutional practice, baseline MRI imaging is usually only obtained if there is a concern for T4 disease. Further studies are needed to evaluate adaptive planning and other techniques with reference to accepted IMRT techniques [53,68].

## 11. Hematologic and Genitourinary Toxicity: Implications for Treatment Planning

Advances are also being made in hematologic toxicity amongst patients receiving chemoradiation for anal cancer. A recent study categorized total pelvic bone marrow into 3 subsites (lumbosacral bone marrow, including the entire sacrum and L5 vertebral body; iliac bone marrow extending from the iliac crests to the superior border of the femoral head; and lower pelvic bone marrow, including the pubic bones, ischia, acetabula, and proximal femurs) and used a generalized linear model to assess the correlation between the equivalent uniform dose to individual pelvic subsites and hematologic outcomes [117]. The analysis suggests that radiation to lumbosacral bone marrow, total pelvic bone marrow, and iliac bone marrow were individually associated with hematologic toxicity [117]. Another study found that treatment of the pelvic bone marrow to a mean dose of ≥30 Gy was associated with a 14-fold increase in the odds of developing grade 3+ hematologic toxicity (compared to <30 Gy), supporting the role of mean dose to the pelvic bone marrow as a useful predictor for hematologic toxicity [118]. A retrospective study suggested that smaller volumes of the pelvic bone marrow were correlated with lower 3-week blood counts [119]. Sparing ≥750 cc of pelvic bone marrow from doses of ≥30 Gy was associated with 0% grade 3+ leukopenia or neutropenia at week 3; furthermore, higher V40 Gy to the lower pelvic bone marrow was associated with cytopenia [119]. The authors suggest that sparing a critical marrow reserve and limiting lower pelvis V40 may reduce hematologic toxicity risk [119].

Recent studies have also reported efforts to mitigate hematologic toxicity through selective avoidance of bone marrow with ^18^FDG-PET guidance [120,121]. In a study of ten patients with locally advanced anal cancer, Franco et al. outlined pelvic bone marrow defined as either the whole outer contour of pelvic bones or as active bone marrow identified using ^18^FDG-PET. The authors demonstrate a degree of reduction in dose to active pelvic bone marrow defined using ^18^FDG-PET compared to optimization using iliac crests per RTOG 05-29, mirroring the reduction in dose when accounting for pelvic bone marrow outlined by the outer surface of external bony structures [120]. A single-arm prospective phase II study evaluated ^18^FDG-PET and found that ^18^FDG-PET-guided IMRT with bone marrow sparing was associated with a reduction in acute hematologic toxicity (19% experienced ≥G3 acute hematologic toxicity) [121]. These findings are exciting as they suggest further means through which hematologic toxicities may be reduced in patients with anal cancer; efforts are needed to identify patient populations most likely to benefit from the incorporation of ^18^FDG-PET in IMRT planning [120,121].

A retrospective cohort of 95 women with anal cancer assessed the incidence of vaginal stenosis after definitive chemoradiation. The study demonstrated 21.4% with grade 0 vaginal stenosis, 14.3% with grade 1, 27.1% with grade 2, and 37.1% with grade 3 [122]. Younger age, greater tumor dose, and earlier treatment year were associated with a higher grade of vaginal stenosis [122]. Furthermore, current atlas-defined female sexual organs at risk do not specifically include the clitoris, and the impact of radiotherapy on the clitoris and late sexual toxicity is poorly understood [123]. Recent evidence supports the feasibility of contouring the anatomically accurate clitoris on standard planning imaging and demonstrates that clitoris-sparing vs. standard IMRT can reduce clitoral dose, suggesting possible safe reduction in sexual toxicity compared to standard IMRT [123].

Notably, the prospective DILANA trial is designed to evaluate the incidence and grade of vaginal fibrosis and to reduce vaginal wall dose in women receiving IMRT for anal cancer. DILANA will randomize patients to two arms that differ only in the diameter of the tampon used during treatment and will assess the incidence and grate of vaginal fibrosis 12 months after chemoradiation [124].

## 12. Timing of Treatment Evaluation and Persistent/Recurrent Disease

Full regression of anal cancer often takes weeks to months after the completion of chemoradiotherapy; follow-up usually entails assessment at four-week intervals using digital rectal exam and anoscopy at 3 months after completion of therapy. Biopsy is used in patients with residual disease at 6 months or progressive tumor at any time.

In RTOG 87-04, treatment response was evaluated with biopsies 6 weeks after therapy. In their cohort, 12.0% had positive biopsy results [48]. Salvage chemoradiotherapy consisted of a radiation boost to an additional 9 Gy to the region of residual disease and one dose of 5FU and cisplatin. Of patients who received salvage chemoradiotherapy, complete response was achieved in 55%; it was uncertain whether patients treated with salvage chemoradiotherapy were slow or nonresponders [48].

Slow response to chemoradiotherapy may lead some patients to have unnecessary surgery. A post-hoc analysis of patients in ACT II suggests that many patients who do not experience a clinical complete response at 11 weeks after treatment eventually respond by 26 weeks [125]. In this post hoc analysis, of those who underwent assessments at 11 weeks, 18 weeks, and 26 weeks after completion of primary treatment, complete response was achieved in 52% at week 11, 71% at week 18, and 78% at week 26 [125]. Among patients who completed all 3 assessments, rates of 5-year overall survival of patients who achieved complete response at 11 weeks, 18 weeks, and 26 weeks were 85% (95% CI, 81–88%), 86% (95%, 82–88%), and 87% (95% CI, 84–90%), respectively, supporting assessment at 26 weeks post-chemoradiotherapy but also highlighting the length of time some patients may need to achieve complete response [125].

## 13. Treatment of Persistent/Recurrent Disease

Despite generally good outcomes after primary chemoradiation without surgery for primary anal cancer, consistent with the ACT II results [125], studies demonstrate persistent disease in 10–15% of patients [28,126,127,128]. The primary treatment for persistent disease is APR [129]. In the UKCCCR trial comparing chemoradiotherapy vs. radiotherapy alone, patients who experienced <50% response underwent salvage APR, of whom 60% were free of locoregional recurrence [44]. In RTOG 87-04, after salvage chemoradiotherapy, 9 of 10 patients with persistent disease underwent APR, of whom 6 recurred [48].

Furthermore, among patients who received primary chemoradiotherapy for anal cancer, multiple retrospective series show that 10–30% of patients may experience recurrent disease [72,128]. Similar to persistent disease, salvage APR is a potentially curative option for locally recurrent anal cancer with 5-year overall survival of 40–60% [69,128,130,131,132]. Other series report survival of over 80% [128,133,134,135,136].

Efforts have been made to identify differences in outcome comparing persistent vs. recurrent disease [128]. Although some studies demonstrate no survival difference among patients who receive salvage APR for persistent vs. recurrent disease [135,137,138], others suggest that persistent disease or early recurrence may be associated with worse outcomes, possibly due to more aggressive tumor biology in persistent disease or early recurrence [132,133,139,140]. Innovations in surgical technique for APR may improve outcomes [131]. However, surgical morbidity is high, and wound complications occur in upwards of 80% in some series [141,142]. Systemic therapy remains the standard of care for unresectable recurrent disease, with ongoing efforts to identify regimens that can improve outcomes [143,144,145,146,147,148,149].

Treatment of locally recurrent anal cancer with external beam re-irradiation and/or intra-operative radiotherapy (IORT) is merits further study [128]. A cohort of 10 patients with locally recurrent anal cancer who received at least 30 Gy to the primary lesion received an additional 27–45 Gy re-irradiation for recurrent disease [150]. Three patients who received re-irradiation without surgery were disease-free at a median of 84 months’ follow-up, and of five patients who received re-irradiation and subsequent surgery, three were disease-free at a median of 43 months’ follow-up [150].

At our institution, IMRT and/or proton radiotherapy are considered when re-irradiating locally recurrent disease, with treatment delivered to gross disease only [128]. A report of patients with locally recurrent anal cancer treated with IORT found that although 50% demonstrated positive resection margins, fewer than a quarter experienced recurrence within the IORT treatment field, suggesting a benefit from IORT [129]. Another study of 14 patients with locally recurrent anal cancer who received salvage surgery and IORT did not find evidence of improved survival or local control associated with IORT [151]. Further work is needed to clarify treatment strategies in the setting of locally recurrent disease.

## 14. Proton Therapy in Anal Cancer

**Figure 3:**
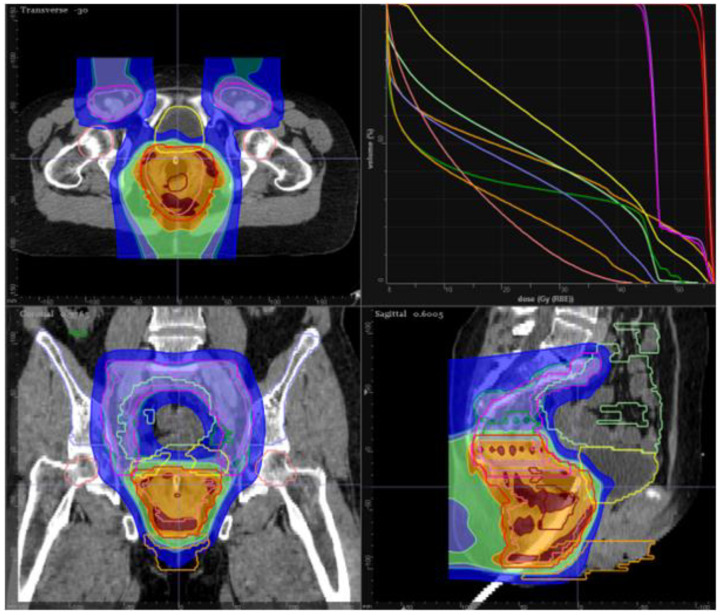
Example of pencil beam scanning proton plan for patient with early stage anal cancer. Elective inguinal nodes were treated with an AP field to 42 Gy. Elective upper pelvic nodes were treated to 45 Gy. Primary tumor and mesorectum treated to 50.4 Gy in 28 fractions.

There has been growing interest in the potential for proton RT to decrease the risk of acute and late adverse effects associated with treatment. Proton therapy facilitates delivery of energy at defined depths, allowing low entry dose and minimal exit dose, theoretically decreasing treatment adverse effects [152,153]. Various retrospective series and dosimetric studies suggest that protons can decrease hematologic toxicity in patients with gastrointestinal cancers, which may benefit patients with anal cancer undergoing concurrent chemotherapy [54,152,154,155,156]. Notably, dosimetric analyses suggest pencil beam scanning proton therapy may improve dose delivery and improve sparing of non-target structures compared to IMRT and 3DCRT (Figure 3) [54,155].

A recent multi-institutional prospective feasibility study assessed concurrent chemoradiation with pencil beam scanning proton therapy with 5FU and MMC in patients with clinical T1-4, N0-3 anal cancer [54]. The rate of grade 3+ radiation dermatitis in the study cohort was 24%, compared to 48% in RTOG 98-11 [59]; the 2-year rate of overall complete response was 88% [54]. These data support the feasibility of pencil beam scanning proton therapy for anal cancer. An ongoing study is assessing linear energy transfer-optimized intensity modulated proton therapy (LET-IMPT) with cisplatin and 5FU, with primary endpoint defined as physician-reported grade 3+ gastrointestinal, genitourinary, and hematologic toxicity (NCT03690921). Another ongoing trial is assessing proton beam radiotherapy (50.4–54 Cobalt Gray Equivalent [CGE] in 28–30 fractions), with primary outcome defined as 3-month grade 3+ hematologic, gastrointestinal, genitourinary, and dermatologic toxicity (NCT03018418). At our institution, we also consider protons to minimize toxicity in the setting of re-irradiation for persistent/recurrent disease [128].

## 15. Ongoing Trials and Studies in Development: PLATO (ACT III, IV, V)

Newer trials are attempting to elucidate ways in which therapy can be further personalized for each patient. Because more advanced lesions (T3-4 and node-positive) have higher rates of treatment failure, the United Kingdom’s Personalising Anal Cancer Radiotherapy Dose (PLATO) trial portfolio, which includes ACT III, ACT IV, and ACT V, examines risk-adapted therapy and stratifies early-stage and late-stage disease [76]. The trials have as their primary endpoint rates of 3-year locoregional failure.

As the Nigro protocol demonstrated effective control of early-stage disease with doses as low as 30 Gy [22], the nonrandomized phase II study ACT III will evaluate dose-reduced chemoradiotherapy in patients who underwent local excision and were found to have T1N0 disease. Patients with >1 mm margin will undergo observation and patients with ≤1 mm margin will be treated with chemoradiotherapy (41.4 Gy in 23 fractions with capecitabine) [157].

In the phase II ACT IV study of patients with intermediate-risk disease, patients with cT1-T2 (≤4 cm) node negative disease will be randomized to the standard radiation arm of 50.4 Gy in 28 fractions or the dose de-escalated arm of 41.4 Gy in 23 fractions. ACT IV will assess if similarly excellent rates of locoregional control with lower rates of adverse effects are achievable with the de-escalated dose [157].

Lastly, the phase II/III ACT V trial, which includes patients with locally advanced disease with tumors >4 cm or positive nodes, will evaluate radiation dose escalation to 53.2 Gy, 58.8 Gy, or 61.6 Gy (28 fractions for all, with standard concurrent chemotherapy), comparing rates of local control and adverse effects. During interim analysis, one of the dose-escalated arms (58.8 Gy or 61.6 Gy) will proceed to phase III. Targeted agents in combination with chemoradiotherapy are also being assessed [158,159,160], as well as vaccine therapy targeted at cells infected with HPV [161,162].

## 16. Immuno-oncology in the High-Risk and Metastatic Setting

Although the majority of patients with anal cancer are treated curatively with chemoradiation, approximately 25% develop distant metastases [163,164]. Historically, these patients were commonly treated with doublet chemotherapy with cisplatin and fluorouracil, based on small retrospective series [165]. Given the association between anal cancer and immunogenic HPV oncoproteins, and tumoral expression of PD-L1 that mitigates the antitumor immune response [166,167], a recent study therefore assessed the role of nivolumab, an anti-PD-1 monoclonal antibody, in patients with metastatic anal cancer [55]. The phase II trial found that of 37 patients who received at least one dose of nivolumab, 9 (24%) demonstrated tumor response, of whom 2 experienced complete response [55].

An ongoing phase III trial is assessing the role of nivolumab after combined modality therapy among patients with high-risk stage II-IIIB anal cancer (NCT03233711). Patients will be randomized to up to 6 months of adjuvant nivolumab vs. observation after combined modality therapy, with the primary endpoint of disease-free survival. Ongoing trials assessing radiotherapy regimens, proton therapy, and immune-oncology are summarized in Table 2.

## 17. Conclusions

Advances in radiotherapy for anal cancer have put chemoradiation at the forefront of primary management for this disease. The advent of IMRT has improved our ability to deliver a conformal dose, affording excellent rates of local tumor control while minimizing adverse effects. Nevertheless, efforts are needed to ascertain doses and treatment delivery strategies that can further improve local control while minimizing toxicity. The results of studies assessing dose schedules that vary based on the extent of disease as well as other treatment strategies such as VMAT and proton therapy are eagerly awaited.

## Figures and Tables

**Table 1:** Key Completed Trials.

Trial	Inclusion	Design	Treatments	Results
Radiotherapy vs. chemoradiotherapy				
UKCCCR Anal Cancer Trial (ACT I) [44,45]	Localized and metastatic, barring exclusion criteria (e.g., previous treatment, cancer at another site, or tumor considered suitable for local excision only [T1 N0])	585 patients; 295 in the CRT arm and 290 in the RT-only arm	45 Gy EBRT and 15 Gy EBRT boost or 25 Gy brachytherapy boost, with vs. without concurrent MMC and 5FU	42-month follow-up: CRT vs. RT alone locoregional recurrence relative risk 0.54 (95% CI 0.42–0.69, *p* < 0.0001) Anal cancer-specific mortality relative risk 0.71 (95%CI 0.53–0.95, *p* = 0.02) 12-year follow-up: for every 100 patients treated with CRT, 25.3 fewer patients experienced locoregional recurrence and 12.5 fewer experienced anal cancer-specific death compared to the RT-only cohort
EORTC [46]	T3-4N0-3 or T1-2N1-3 anal cancer	110 randomized, 103 eligible, 51 to CRT and 52 to RT alone	45 Gy EBRT with 15 Gy or 30 Gy EBRT boost with vs. without concurrent 5FU and MMC	5-year local control greater for CRT vs. RT alone (68 vs. 51%, *p* = 0.02) 3-year overall survival similar between groups (65 vs. 72%, *p* = 0.17)
Omission of MMC				
RTOG 87-04/ECOG 1289 [48]	Patients with any epidermoid malignancy of the anal canal in which the primary tumor was measurable (any T or N stage)	310 randomized, 295 eligible, 145 to EBRT + 5FU, 146 EBRT + 5FU + MMC	45–50.4 Gy EBRT with 5FU with vs. without MMC	MMC associated with greater colostomy-free survival (71% for 5FU and MMC vs. 59% for 5FU alone; *p* = 0.014) and improved disease-free survival (73% for 5FU and MMC vs. 51% for 5FU alone; *p* = 0.0003) No OS difference and greater toxicity with MMC
Cisplatin vs. MMC				
RTOG 98-11 [7,49]	T2-4NanyM0 (T1 or M1 excluded)	682 randomized, 649 eligible, 325 to RT + 5FU/MMC and 324 to RT + 5FU/cisplatin	45 Gy with allowance for 10–14 Gy boost with 5FU + MMC vs. RT + 5FU + cisplatin	5-year colostomy-free survival improved in MMC arm (72 vs. 65%, *p* = 0.05); DFS improved in MMC arm (67.8 vs. 57.8%, *p* = 0.006); OS improved in MMC arm (78.3 vs. 70.7%, *p* = 0.026)
ACT II [50]	Any T, any N, no distant metastases	940 randomized, 472 in RT + 5FU/MMC cohort and 468 in RT + 5FU/cisplatin cohort	50.4 Gy with continuous 5FU, with bolus cisplatin or MMC	3-year colostomy-free similar (68% in MMC arm, 67% in cisplatin arm, *p* = 0.94); DFS similar (69% in both arms, *p* = 0.63); OS similar (79% in MMC arm, 77% in cisplatin arm, *p* = 0.7)
RT dose escalation vs. de-escalation				
RTOG 92-08 [36,48,51]	Any except T1N0	Single-arm phase II study with 47 patients: standard chemotherapy (5FU/MMC) + high dose RT	2 weeks of RT, then mandatory gap, total RT dose 59.4 Gy	Median follow-up duration 12 years, estimated 5-year DFS 53%; estimated 5-year colostomy-free survival 58%; estimated 5-year OS 85%
ACCORD-03 [52]	Patients with tumors ≥ 40 mm, or <40 mm and N1-3M0	2 × 2 factorial randomization: neoadjuvant chemotherapy and CRT (5FU/cisplatin) +/− high-dose RT; 283 of 307 achieved full treatment	45 Gy/25 fractions with standard dose boost (15 Gy) vs. high-dose boost (20–25 Gy) with EBRT or brachytherapy	Similar colostomy-free survival (the primary endpoint) for standard vs. escalated boost dose (78 vs. 74%, *p* = 0.067); nonsignificant improvement in 5-year local control rate for escalated boost dose (83.1%) vs. standard-boost (78.2%)
Intensity-modulated radiotherapy (IMRT)				
RTOG 05-29 [53]	T2N0, T3-4N0-3	Phase II trial evaluating CRT with concurrent 5FU/MMC and dose-painted IMRT	T2N0: 42 Gy elective nodal and 50.4 Gy anal tumor PTVs in 28 fractions T3-4N0-3: 45 Gy elective nodal and 50.4 Gy < 3 cm or 54 Gy > 3 cm metastatic nodal and 54 Gy anal tumor PTVs in 30 fractions	Dose-painted IMRT associated with reduced grade 3+ genitourinary and gastrointestinal toxicity (22 vs. 36%, *p* = 0.014), and grade 3+ dermatologic toxicity (20 vs. 47%, *p* < 0.001) when compared to the historical RTOG 98-11 MMC arm
Pencil beam scanning proton beam radiotherapy (PBS-PT)				
MGH prospective series [54]	T1-4, N0-3 disease	25 patients, of whom 23 completed treatment per protocol	PBS-PT per RTOG 0529 dose schema and concurrent 5-FU/MMC	Grade 3+ radiation dermatitis rate 24%; overall rate of clinical complete response was 88%; 2-year local failure rate 12%, colostomy-free survival 72%, progression-free survival 80%, and overall survival 84%
Immunooncology				
Multicenter US prospective series [55]	Patients with anal cancer (squamous cell only, adenocarcinoma excluded) and at least one previous systemic therapy for surgically unresectable or metastatic disease	Phase II study of 37 patients with metastatic disease	Nivolumab IV every 2 weeks (3 mg/kg)	Of 37 patients who received at least one dose of nivolumab, 9 (24%) demonstrated tumor response, of whom 2 experienced a complete response

**Table 2:** Ongoing Trials.

Trial/NCT ID	Inclusion	Design	Treatments
RT dose escalation vs. de-escalation			
ECOG-DECREASE [75]	T1-2N0M0	Randomized phase II: standard-dose CRT vs. de-intensified CRT	28 fractions vs. de-intensified 20–23 fractions of IMRT with MMC and 5FU or capecitabine Standard: T1-T2 N0: 50.4 Gy to primary tumor with 42 Gy to elective nodal regions, all in 28 fractions De-intensified: T1 N0: 36 Gy to primary tumor with 32 Gy to elective nodal regions, all in 20 fractions T2 N0: 41.4 Gy to primary tumor with 34.5 Gy to elective nodal regions, all in 23 fractions
ACT III [76,157]	T1N0	Single-arm phase II: dose reduced CRT	No RT for >1 mm margin; for <1 mm margin, 41.4 Gy in 23 fractions
ACT IV [76,157]	T1-2, N0	Randomized phase II: standard chemotherapy (5FU/MMC) and standard vs. de-intensified RT	Standard RT arm of 50.4 Gy in 28 fractions or de-intensified radiation arm of 41.4 Gy in 23 fractions
ACT V [76,157]	T3-4, N0-X	Randomized phase II/III: Standard chemotherapy (5FU/MMC) with standard vs. 2 escalated radiation doses	53.2 Gy, 58.8 Gy, or 61.6 Gy all in 28 fractions with standard concurrent chemo. One of the dose-escalation arms will proceed to phase III
Proton therapy			
NCT03690921 (MDACC)	Non-metastatic disease	Single-arm phase II trial assessing adverse effects of proton RT and standard chemotherapy (cisplatin and 5FU)	Linear energy transfer (LET)-optimized intensity-modulated proton therapy (IMPT)
NCT03018418 (Cincinnati)	T2-4 disease with any N	Prospective pilot study evaluating the feasibility of intensity-modulated proton therapy in reducing RT toxicity	Primary target volume 50.4–54 CGE in 28–30 fractions; nodal volumes 42–54 CGE in 28–30 fractions, with 5FU and MMC
NCT04462042 (Umeå University/Sweden)	T2 (>4 cm)-4, N0-1c, M0	Open label, multi-center, randomised phase II study, comparing proton to photon RT	Photon: primary tumor and nodal metastases >2 cm 57.5 Gy in 27 fractions (VMAT/IMRT/tomotherapy); nodal metastases up to 2 cm will receive 50.5 Gy in 27 fractions; elective nodes will receive 41.6 Gy Proton: spot scanning, total dose to the primary tumor target and node metastases >2 cm is 57.5 Gy(RBE) in 27 fractions; nodal metastases up to 2 cm will receive 50.5 Gy(RBE) in 27 fractions; elective nodes will receive 41.6 Gy(RBE)
Immuno-oncology			
NCT03233711	stage IIB (T3N0M0 only), IIIA (T2N1M0), IIIB (T4N0M0), or IIIC (T3N1M0, T4N1M0) invasive squamous cell carcinoma of the anus or anorectum	Randomized phase III trial of nivolumab after combined modality therapy	Up to 6 months of nivolumab IV vs. up to 6 months of observation
NCT02919969	Metastatic anal cancer with no limitations to prior treatment	Phase II study of pembrolizumab	Pembrolizumab 200 mg IV infusion every 3 weeks
NCT03519295	Unresectable locally advanced, recurrent, or metastatic squamous cell anal carcinoma	mDCF (docetaxel, cisplatin, 5FU) with vs. without atezolizumab	8 cycles of mDCF with vs. without MPDL3280A (atezolizumab) for 12 months
NCT04230759 (RADIANCE trial)	Locally advanced disease (IIB: T3N0M0; IIIA: T1-2N1M0; IIIB: T4N0M0; IIIC: T3-4N1M0; T2 > 4 cm Nany)	Phase II trial assessing the efficacy of durvalumab in combination with CRT with MMC + 5FU	53.2–58.9 Gy with nodal and elective nodal irradiation, with MMC + 5FU, with vs. without 12 doses of durvalumab
NCT03944252	Progression on or after first-line systemic therapy for surgically unresectable or metastatic disease	Randomized Phase II trial of cetuximab and avelumab or avelumab alone for unresectable, locally advanced, or metastatic anal cancer progressed after at least one line of systemic therapy	Avelumab IV with vs. without cetuximab, given until progression of disease
NCT04444921	Inoperable, recurrent, or metastatic disease	Randomized phase III trial of nivolumab with chemotherapy in treatment-naive metastatic anal cancer	Carboplatin and paclitaxel with vs. without nivolumab
NCT01285778	T2-4, any N	Phase II assessing the efficacy and toxicity of radiotherapy with 5FU, MMC, and panitumumab	Radiation therapy will be administered concurrent with chemotherapy and panitumumab treatment (IV over 8 weeks)
NCT04472429 (POD1UM-303/InterAACT 2)	Inoperable locally recurrent or metastatic SCAC with no prior systemic therapy other than chemotherapy administered with radiotherapy as a radiosensitizer	Phase III double-blind randomized trial of carboplatin-paclitaxel with Retifanlimab or placebo in patients with inoperable locally recurrent or metastatic disease with no prior systemic chemotherapy	Carboplatin, paclitaxel, and either placebo or retifanlimab

## Data Availability

No new data were created or analyzed in this study. Data sharing is not applicable to this article.
